# Green synthesis of gold nanoparticles using *Pelargonium Graveolens *leaf extract: characterization and anti-microbial properties (An in-vitro study)

**DOI:** 10.12688/f1000research.150769.1

**Published:** 2024-06-04

**Authors:** Ahmed Yousif Mahdi Asker, Aseel Haidar M.J. Al Haidar

**Affiliations:** 1 Department of Pediatric and Preventive Dentistry, University of Baghdad, Baghdad, Iraq

**Keywords:** gold nanoparticles, Pelargonium graveolens, Streptococcus Mutans, Candida Albicans

## Abstract

**Background:**

In recent years, there has been a notable increase in the level of attention devoted to exploring capabilities of nanoparticles, specifically gold nanoparticles AuNPs, within context of modern times. AuNPs possess distinct biophysical properties, as a novel avenue as an antibacterial agent targeting Streptococcus Mutans and Candida Albicans. The aim of this study to create a nano-platform that has the potential to be environmentally sustainable, in addition to exhibiting exceptional antimicrobial properties against Streptococcus Mutans as well as Candida Albicans.

**Methods:**

this study involved utilization of *Pelargonium Graveolens* leaves extract as a cost effective and environmentally sustainable approach for the green synthesis of AuNPs. Subsequently, physicochemical characteristics were assessed employing a variety of analytical methods, including as transmission electron microscopy, X-ray diffraction, Field Emission Scanning Electron Microscope, Zeta potential, Ultraviolet visible absorption spectroscopy, and Energy dispersive X-ray spectroscopy and Fourier transform infrared spectroscopy. The antimicrobial efficacy against Streptococcus Mutans and Candida Albicans was evaluated. Nanoparticles of various shapes, including hexagonal, spherical, semi-spherical, and triangular, were synthesized. These nanoparticles exhibited a mean particle size of 294nm and demonstrated low degree of aggregation. These nanoparticles exhibited long-term stability and were capable of facilely combining with diverse bioactive compounds.

**Results:**

The study demonstrated that AuNPs which is synthesized by green methods display potent antimicrobial properties.

**Conclusion:**

Utilization of Pelargonium Graveolens AuNPs may exhibit a promising potential as an antibacterial agent against Streptococcus Mutans and Candida Albicans. Nanoparticles (NPs) have the potential to serve as a novel approach for addressing pathogen infections as well as for biomedical, dental and pharmaceutical purposes in the future.

**Abbreviations**
ANOVA: One-way Analysis of Variance
EDX: Energy Dispersive X-ray Spectroscopy
FESEM: Field Emission Scanning Electron Microscope
FTIR: Fourier Transform Infrared
MIC: Minimum Inhibitory Concentration
SD: Standard Deviation
SE: Standard error
TEM: Transmission Electron Microscopy
UV-Vis: Ultraviolet visible absorption spectroscopy
XRD: X-ray Diffraction

## Introduction

Nanotechnology has recently provided novel options for the treatment of several ailments. There has been much research’s concerning gold nanoparticles GNPs are typically regarded to be biocompatible for medical usage as the antioxydant, anticancer, drug delivery and antimicrobial agents. ^1^^–^^4^ The comprehensive investigation of gold nanoparticles has surpassed that of other metal nanoparticles, owing to their comparatively minimal toxicity towards animal and microorganism cells in comparison to other metal nanoparticles. ^5^ Gold nanoparticles display unique physicochemical characteristics in comparison to bulk solids, owing to their significantly larger surface area relative to their volume. ^6^ The coloration of Gold nanoparticles is dependent on their structural characteristics, size, and level of aggregation. The application of red colloidal gold has been employed as a means of regeneration therapy. In addition, gold nanoparticles have been utilized in diagnostic and therapeutic drug delivery applications. The utilization of microorganisms, enzymes, and plant extracts has been proposed as a viable and environmentally conscious substitute for conventional chemical and physical techniques in the production of metallic nanoparticles. The utilization of the green synthesis approach has attracted significant interest as a cost-effective and eco-friendly methodology that facilitates the production of nanoparticles of diverse shapes and sizes, Demonstrating remarkable stability and compatibility. ^7^ The study done by Lee et al. investigates the efficacy of distinct active compounds derived from plants, particularly is flavones, protocatechuic acid, and Gallic acid, as reducing agents for the green synthesis of modified gold nanoparticles (AuNPs). The resulting nanoparticles exhibited remarkable stability and biocompatibility, with a lifespan of up to three months. ^8^ In general, it has been observed that HAuCl _4_ forms chlorine-carbon bonds with plant extracts. ^9^ Furthermore, the plant extracts contain starch and glucose constituents that can serve as stabilizing and reducing agents during the synthesis of gold nanoparticles. This enables the nanoparticles to be stored for an extended period of 17 months while maintaining their stability. ^10^ Shankar et al in 2003 were able to synthesize gold nanoparticles (GNPs) of varying shapes through the utilization of Pelargonium graveolens leaves and its entophytic fungus. The plant Pelargonium graveolens and its entophyte have been recognized as potential candidates for the synthesis of gold nanoparticles. In particular, the researcher had conducted research on the reduction of aqueous chloroaurate ions using the leaves of the Pelargonium graveolens plant and an entophytic fungus. ^11^ Due to its, antioxidant, antibacterial, antifungal, hypoglycemic, anti-inflammatory, and cancer-fighting were only some of the reported biological and pharmacological effects of Pelargonium graveolens. Extracts from this plant are useful because they contain bioactive compounds. Citronellal, geranial, and linalool are three major bioactive components found in Pelargonium graveolens, which may explain their properties. In light of this, it is now recommended in herbal medicine to investigate the chemical components of this plant. In order to create safe and effective medications, it is crucial to understand the scientific foundation of their therapeutic activities. ^12^ Nanoparticles hold distinct promise compared to their bulk equivalents. Since they are small and have a large surface area, they have demonstrated significant biological activity in the human body. ^13^ The use of nanotechnology in dentistry has significant potential for transformative advancements. Nevertheless, it is crucial to acknowledge the potential risks associated with its misuse. The prioritization of applications within this discipline will ultimately be determined by factors such as time and human requirements. ^14^ In recent years, there has been a significant increase in the utilization of nanoparticles in the formulation and advancement of various dental materials. This is mostly due to their ability to provide a distinctive amalgamation of characteristics. The nanoparticles have a relatively large surface area to volume ratio in comparison to particles of equivalent substance due to their small size. ^15^ The present study outlines a straightforward procedure for synthesizing AuNPs by employing Pelargonium Graveolens extract as an inexpensive and environmentally benign, stabilizing and reducing agent, furthermore, an evaluation was conducted on the physical characteristics and antimicrobial efficacy towards Streptococcus mutans and Candida albicans.

## Methods

Chloroauric acid (HAuCl _4_.3H _2_O), from (Sigma-USA ^®^^,^^™^). Muller-Hinton agar and nutrient broth medium, from (Accumix-Spain ^®^^,^^™^). Ethanol bought from (Duksan-Korea ^®^^,^^™^), all of the additional chemicals and reagents utilized in the experiments were of analytical grade.

### Microorganism

The study evaluated the antimicrobial effectiveness of the prepared Pelargonium Graveolens AuNPs against clinical isolates of Streptococcus mutans and Candida albicans. These isolates were obtained from the oral cavity of patients in the Pediatric and Preventive Dentistry Department at the University of Baghdad’s College of Dentistry. The laboratory utilized standard biochemical methods to process and identifies these isolates. Following the transfer of the stock cultures onto Mueller-Hinton agar medium, an overnight incubation at 37°C was conducted, followed by storage at 4°C.

### Preparation of Pelargonium Graveolens leaf extract and Green synthesis of Pelargonium Graveolens AuNPs

Pelargonium Graveolens leaves collection in Baghdad Governorate, Iraq. The collected leaves were thoroughly cleansed under running water to eliminate traces of chemicals and grime. After a thorough rinsing, the leaves of Pelargonium Graveolens were incubated overnight at 37 ^o^C to dry. After incubation, the completely desiccated leaves were ground into a fine powder for use in the extraction process. A quantity of 100 g of air-dried ground plant material was subjected to extraction using aqueous alcohol as the solvent (methanol, water, ethanol (1:1:3) 80 % v/v) 500 mL the samples were subjected to Soxhlet extraction for a duration of 8 hours on a water bath. The extracts were subjected to solvent removal under reduced pressure at a temperature of 45°C using a rotary evaporator. Subsequently, the desiccated crude concentrated extracts were measured in order to determine the yield. Following this, the powder was diluted to achieve a concentration of 250 μg/ml, which corresponds to MIC of the plant extract, after that, the extract was filtered 50ml of it was taken prepared for the purpose of using it with gold chlorides to act as a reducing agent in the preparation of gold nanoparticles. The gold ion solution was prepared in accordance with the methodology outlined by Ref. 16. with some modification, one gram of HAuCl _4_ dissolved by 100 mL of DW to form a 10 mg/ml solution. Gold nanoparticles (Au NPs) were synthesized through a mix of a 1 mg/ml HAuCl _4_ gold ion solution and a 50 ml Pelargonium Graveolens extract. The solution was subjected to a magnetic stirrer for duration of 30 minutes while being slightly heated to a temperature range of 35°C to 45°C. As a result, the mixture exhibited a quick alteration in color, transforming into a dark shade of purple/red within a brief time span. The observed change in color can be assigned to the formation of gold nanoparticles (AuNPs) with a concentration of 15.7 (ppm). The manifestation of a purple color following the mixture of the plant extract and HAuCl _4_ solution signifies the production of Pelargonium Graveolens AuNPs.

### Characterization of *Pelargonium Graveolens* AuNPs

An X-ray diffract meter (XRD-6000, SHEMADZU-Japan ^®^^,^^™^) used to evaluate the produced nanoparticles characteristics. (Current = 30 mA; voltage = 40 kV). A Cu-Kα incident beam (λ = 1.542 A°) at 2θ = 20°-80° used to determine the patterns in which the particles diffracted. Transmission electron microscopy (PHILIPS CM-120-USA ^®^^,^^™^) is operating at 100 kV to evaluate the size and morphologies of the nanoparticles. Pelargonium Graveolens AuNPs solutions were characterized using a UV-Vis spectrophotometer (SHEMADZU1200i-Japan ^®^^,^^™^) the experiment employed a double beam UV-Vis spectrophotometer to observe the beams under different conditions within the spectral range of 200–1100 nm. An FTIR analysis was conducted utilizing the PerkinElmer Spectrum-USA instrument, covering spectral ranges from 4500 to 500 cm ^-1^. FTIR analysis was conducted in order to examine the functional groups of biomolecules that are responsible for capping, viable stabilization, and reduction of the prepared Pelargonium Graveolens AuNPs. A Zeta potential test was conducted using a Zetasizer Nano ZS (Malvern Instruments ^®^^,^^™^) at a temperature of 25°C. The (Zetasizer 6.01 software ^®^^,^^™^)was employed for data analysis. The purpose of the zeta-potential measurements was to examine the stability of Pelargonium Graveolens AuNPs in the suspension. Field Emission Scanning Electron Microscopy (FESEM) employed utilizing a (ZIESS, Germany ^®^^,^^™^) Electron Microscopy to identify the morphological features of Pelargonium Graveolens AuNPs. The investigation of elemental composition was carried out using EDX with a (Thermo Scientific TM NORANTM ^®^^,^^™^ System 6 EDS system).

### Biological activities of Pelargonium Graveolens AuNPs

**Antibacterial efficacy of Pelargonium Graveolens AuNPs**

The antimicrobial effect of Pelargonium Graveolens AuNPs was investigated using the Agar diffusion method. Ten isolates of each S. mutans and C. Albican were applied separately on MH agar plates; the previous approach was explained by Ref. 17. Mueller–Hinton agar media was poured separately into sterile petri dishes and wait until setting. A volume of 0.1ml of activated Streptococcus mutans and Candida Albican was evenly distributed onto Mueller-Hinton agar plates and allowed to incubate at the room temperature for duration of 10 minutes. Subsequently, using a sterile stainless steel Cork borer, uniform and sufficiently deep wells measuring 6mm in diameter were generated in the Mueller-Hinton agar. This process was repeated six times on each plate and for each microorganism. Each well was filled with 0.2 mL of the agent to be tested at varying concentrations of 0.06, 0.12, 0.25, and 0.5 mg/mL. The last well filled with Chlorhexidine 0.12% as a positive control, while the center well filled deionized water as a negative control. Plates left at the room temperature for 10 minutes and then incubated aerobically for 24hrs at 37C. Each zone of inhibition was measured across the diameter of each well. No zone of inhibition indicated complete resistant of S. mutans and C. Albican a to the test agent. Then, we calculated the MIC, MBC, and MFC of Pelargonium Graveolens AuNPs against the individual microorganisms by agar dilution method against S. mutans, and C. albicans. Pelargonium Graveolens AuNPs concentrations of 0.06, 0.12, 0.25, 0.5 mg/ml were serially diluted in Mueller–Hinton agar to make 10 mL of agar and extracts, which were then placed into Petri dishes and allowed to solidify before being inoculated with 0.1 ml of activated isolates of microorganisms. All of these Petri dishes, including the control plates, were incubated at 37°C overnight (the negative control, which contained Mueller-Hinton agar with microbial inoculums without the addition of the extracts, and the positive control plates, which contained MH-A and different concentrations of the Pelargonium Graveolens AuNPs without microbial inoculums). The presence or lack of bacteria growth on the plates is checked. The MIC of Pelargonium Graveolens AuNPs is the concentrations at which microbial growth is totally inhibited. The identical approach was followed for all of the microbial isolates, that had been described previously. ^18^ The MBC/MFC of Pelargonium Graveolens AuNPs is the concentrations at which microbial growth is totally killed.

### Statistical analysis

Statistical Package for Social Research was used for descriptive analysis, and presentation (SPSS Statistical Package for social Science version-22, Chicago, Illinois, USA). The Levene test and Shapiro-Wilk test were conducted on a quantitative variable, which encompassed the minimum, maximum, mean, standard deviation (SD), and standard error (SE). The One-way Analysis of Variance (ANOVA) statistical method was employed, along with Tukey’s Honestly Significant Difference (Tukey’s HSD) and Dunnett’s T3 posthoc tests, to conduct multiple pairwise comparisons among the groups. The results are considered statistically insignificant when the P value is greater than 0.05, while they are deemed statistically significant when the P value is less than 0.05.

## Results

### Characterization of prepared nanoparticle

**Determination of the shape and size**

**Figure 1.**
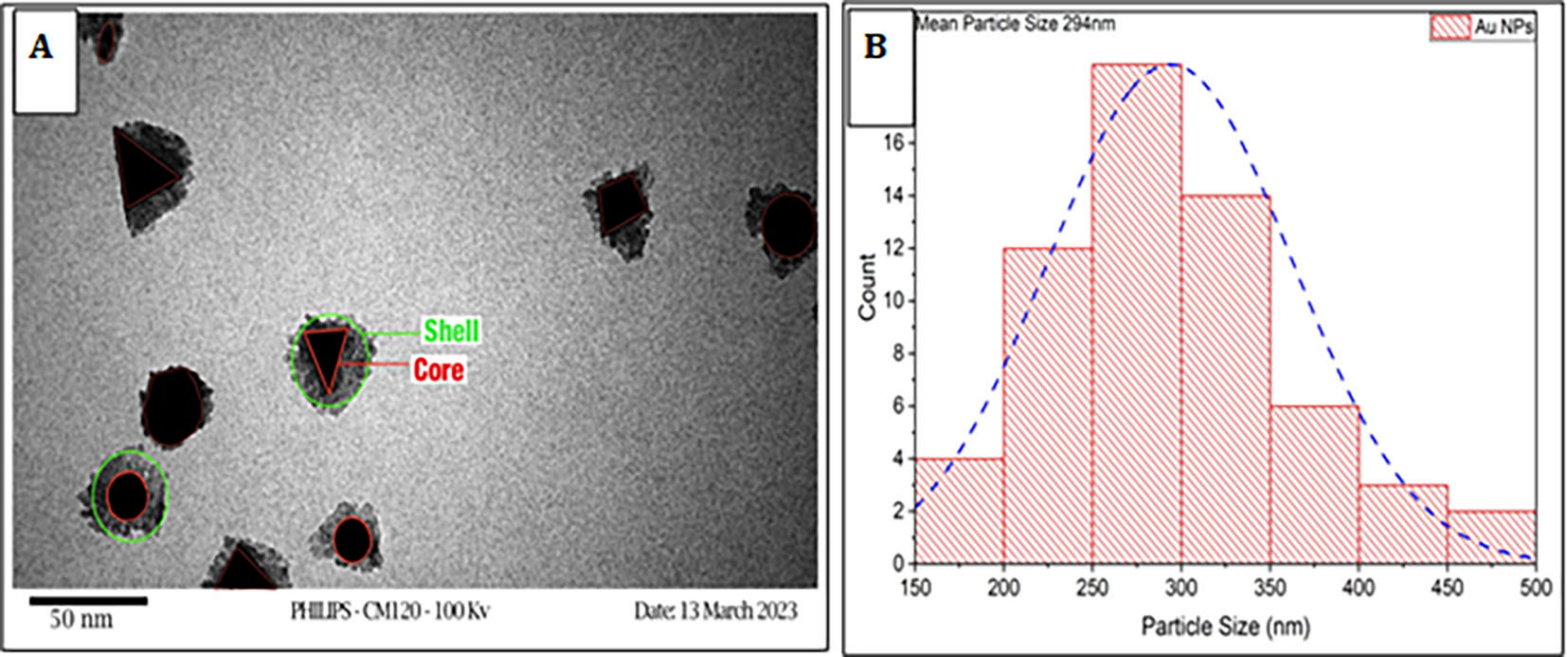
A – TEM of *Pelargonium Graveolens* AuNPs B – Distribution of particle size of *Pelargonium Graveolens* AuNPs.

Transmission electron microscopy (TEM) was employed to investigate the characterization of the synthesized nanoparticles (NPs). The morphology of gold nanoparticles (AuNPs) is characterized by a combination of hexagonal, spherical, semi-spherical, and triangular-shaped particles. The size and abundance of gold nanoparticles particles decrease as the concentration of *Pelargonium Graveolens* leaves extracts increases. The morphology *Pelargonium Graveolens* AuNPs is depicted in Figure 1-A, where the AuNPs are represented as a core and the *Pelargonium Graveolens* acts as a shell surrounding the AuNPs surface, and as shown in Figure 1-B the average size of *Pelargonium Graveolens* AuNPs has been estimated to be 294 nm due to the small concentration of *Pelargonium Graveolens* leaves extracts is 250 μg/ml (MIC of plant extract), It is considered a good and safe particle size because the smaller size easily to uptake by mammalian cells this is agree by Ref. 19.

### XRD analysis

**Figure 2.**
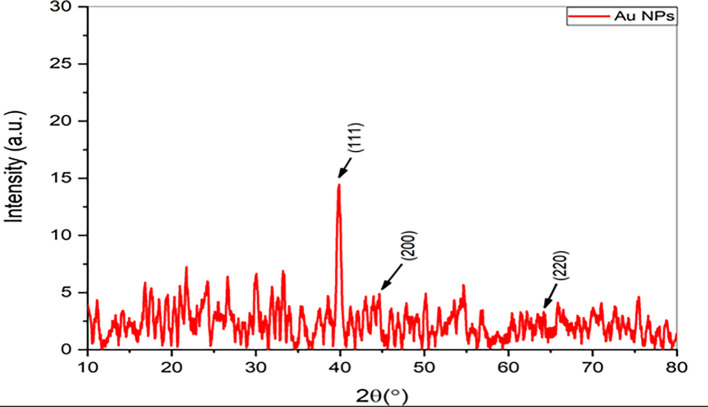
XRD spectra of *Pelargonium Graveolens* AuNPs.

For further determination of the *Pelargonium Graveolens* AuNPs, The X-ray diffraction (XRD) pattern was utilized to analyze the diffraction peaks observed at 2θ = 39.9°, 44°, and 65.28°, which can be attributed to the 111, 200, and 220 crystallographic planes of the cubic structure of the gold nanoparticles (Au NPs) as depicted in Figure 2. These results are consistent with the data provided by the Joint Committee on Powder Diffraction Standards (JCPDS) under the reference number 001-1172. The results mentioned above agree with the data presented by Ref. 20. The current data indicates that XRD pattern of the *Pelargonium Graveolens* AuNPs closely resembles that of pure gold nanoparticles. The mean size of nano crystallites in *Pelargonium Graveolens* AuNPs is 69.587 nm calculated From the well-known Scherrer formula (D = 0.9λ/β. Cos θ) In this context, the symbol D represents the average size of crystallites, β refers to the line broadening as quantified by the full width at half maximum (FWHM) of a peak, λ represents the wavelength of X-rays used for irradiation, and θ signifies the maximum position value of the peak ^21^ as shown in Table 1.

**Table 1.** Crystallite size inside *Pelargonium Graveolens* AuNPs.

2 theta (degree)	hkl	FWHM (deg)	2 theta (rad)	FWHM (rad)	D (nm)
39.8474	111	0.96	0.348	0.017	8.799
44.04	220	0.09	0.384	0.002	95.178
65.28	220	0.09	0.570	0.002	104.783
					69.587

### FTIR spectra of prepared NPs

**Figure 3.**
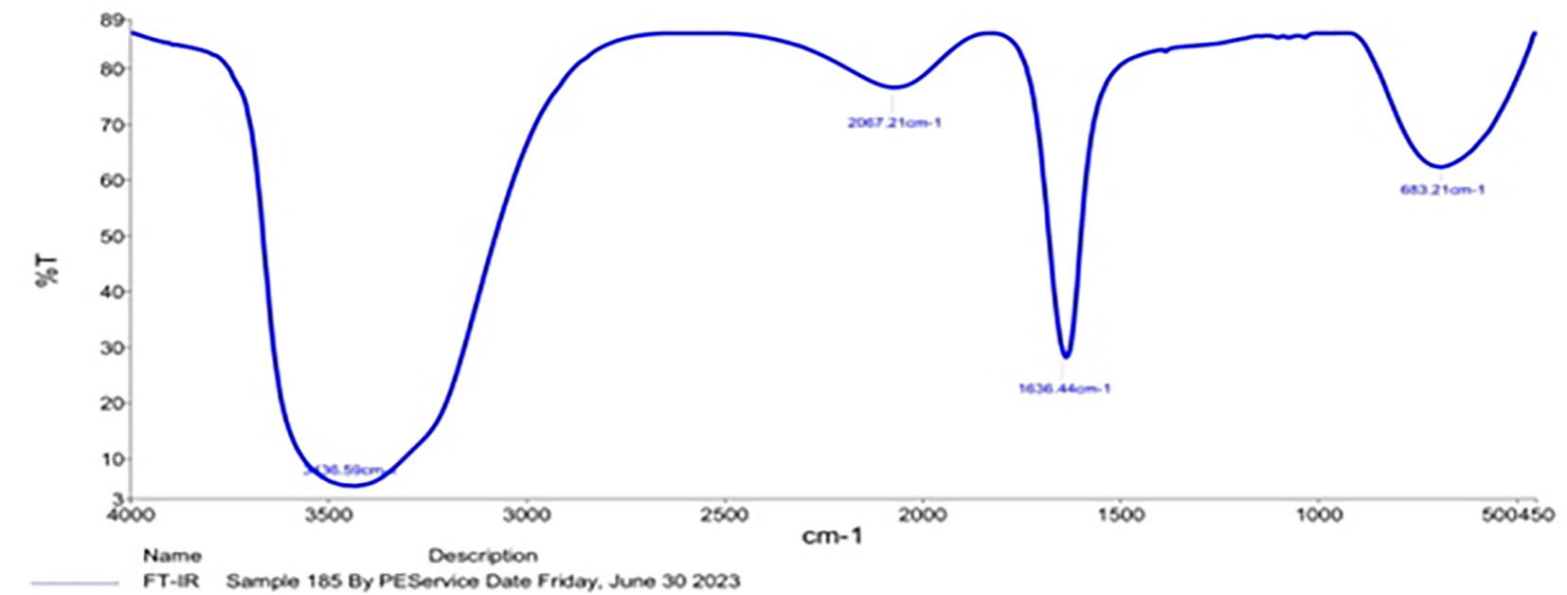
FT-IR details of *Pelargonium Graveolens* AuNPs.

In the cases of *Pelargonium Graveolens* AuNPs the FTIR measurements detailed in Table 2 were conducted in an effort to determine the character of the organic protection layer surrounding the AuNPs based on comparable research in the literature. ^22^^,^^23^ The observed significant shifts in the spectra of certain peaks subsequent to the formation of AuNPs are hypothesized to be attributed to the impact exerted by the adjacent metal surface. Hence, it is postulated that the observed displaced peaks, as documented in Table 2, are indicative of the presence of organic matter surrounding the synthesized AuNPs. Figure 3 demonstrates the Fourier Transform Infrared (FTIR) analysis conducted to assess the significant biomolecules involved in the capping and stabilization of *Pelargonium Graveolens* AuNPs synthesized through a green method. Intense absorptions are observed at 3436.59, 2067.21, 1636.44, and 683.21 cm ^-1^. IR band at 3436.59 cm ^-1^ refers to the OH stretching mechanism of the proteins, polyphenols, and carbohydrates. The band noticed at 2067.21 and 683.21 cm ^-1^ related to the C≡C expanding of the alkynes group and C–N/C–Cl in plane bending, respectively. Furthermore, the IR band at 1636.44 cm ^-1^ is characteristic of the C=O expanding of the carboxylic group. Hence, it is possible that enzymes/proteins are involved in the process of reducing metal ions through the oxidation of aldehydes to carboxylic acids. Proteins possess the capacity to form links with gold nanoparticles (AuNPs) via carboxylate ions found in amino acid residues or through free amine groups within the protein’s structure. ^24^^,^^25^ In addition, the presence of the C=O stretching mode indicates the presence of the carboxylic acid (-COOH) group in the material that is attached to gold nanoparticles (Au NPs). Hence, the spectral peak observed at a wavenumber of 1636.44 cm ^-1^ can be attributed to the manifestation of amine groups, signifying a possibility of protein binding onto gold nanoparticles. The provided information holds significant value in informing the design of functionalization procedures, particularly in the context of utilizing particles for drug delivery purposes.

**Table 2.** FT-IR details of *Pelargonium Graveolens* AuNPs.

Peak number	X (cm ^-1^)	Y (%T)
1	3436.59	4.85
2	2067.21	76.91
3	1636.44	28.12
4	683.21	62.53

### UV-Vis absorbance spectrum

**Figure 4.**
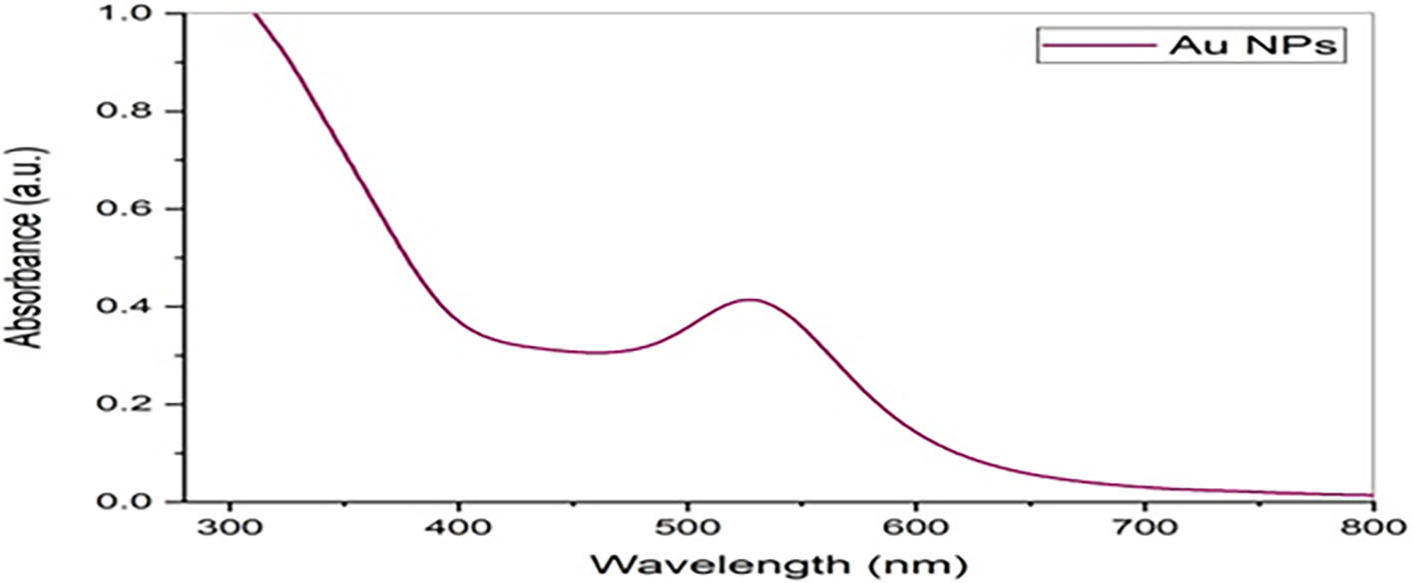
UV-visible absorption spectra of *Pelargonium Graveolens* AuNPs.

Figure 4 shows the absorbance of the peak of *Pelargonium Graveolens* AuNPs was at 527 nm. *Pelargonium Graveolens* AuNPs show a massive peak. The successful synthesis of *Pelargonium Graveolens* AuNPs was confirmed by the observation of a dark purple color in the reaction mixture and the presence of specific absorption spectra at a wavelength of 527 nm in the UV-vis absorption spectroscopy. This outcome aligns with findings reported in previous studies. ^16^

### Zeta potential

**Figure 5.**
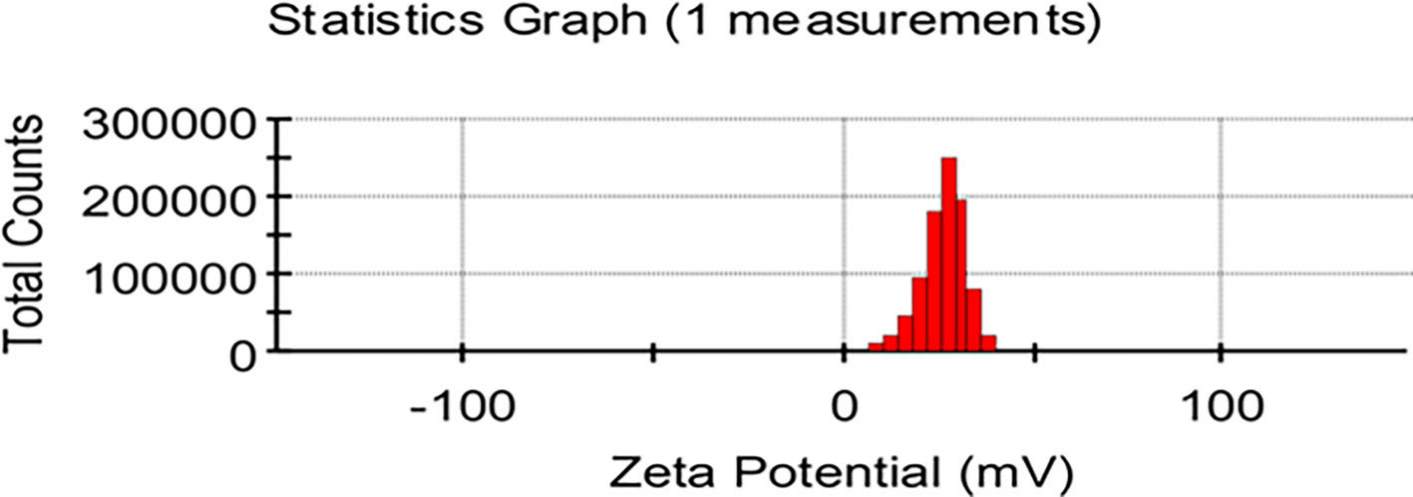
Zeta potential of *Pelargonium Graveolens* AuNPs.

The zeta potentials of the particles present in the sample of *Pelargonium Graveolens* AuNPs were determined at a temperature of 25°C. Figure 5 shows that the *Pelargonium Graveolens* AuNPs sample showed positive zeta-potential values between 0 and +50 mV the observed range can be classified as an initial state of stability for colloidal systems that agree with. ^26^ The zeta potential of the *Pelargonium Graveolens* AuNPs was measured to be 27 mV Figure 5. This value suggests that the nanoparticles were stable and had a reduced propensity to aggregate and form larger particles.

### FESEM-EDX analysis of particle composition

**Figure 6.**
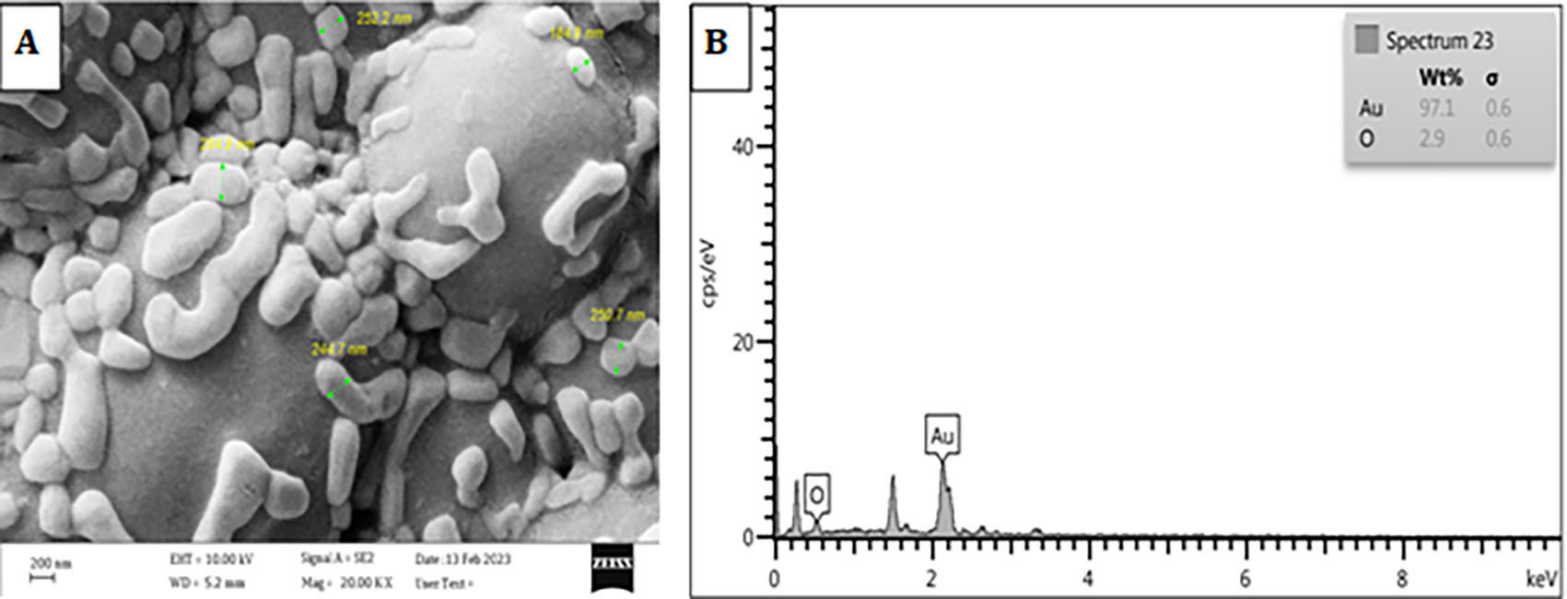
A SEM image of Pelargonium Graveolens AuNPs, B EDS spectra of Pelargonium Graveolens AuNPs.

The results clearly presented in Figure 6-B demonstrate that the particle composition unequivocally corresponds to gold. The presence of trace amounts of additional elements, such as oxygen (O), was observed in the elemental composition of the plant extract after the solvent evaporation process. The elemental composition of the plant extracts was determined using inductively coupled plasma optical emission spectroscopy (ICP-OES), revealing the presence of trace amounts of these elements, among others. The data exhibited a strong correlation between the concentrations of different elements present in the plant extracts and the results obtained from Energy-Dispersive X-ray Spectroscopy (EDX), as seen previously in this report, ^16^ and the findings from field emission scanning electron microscopy (FESEM) analysis, as depicted in Figure 6-A, indicate that the gold nanoparticles (AuNPs) were effectively separated and did not form aggregates. This can be attributed to the presence of *Pelargonium Graveolens*, which functions as a protective agent, corroborating the observations made through transmission electron microscopy (TEM) and this agree with. ^27^

### Biological studies

**Antibacterial activity of *Pelargonium Graveolens* AuNPs**

**Figure 7.**
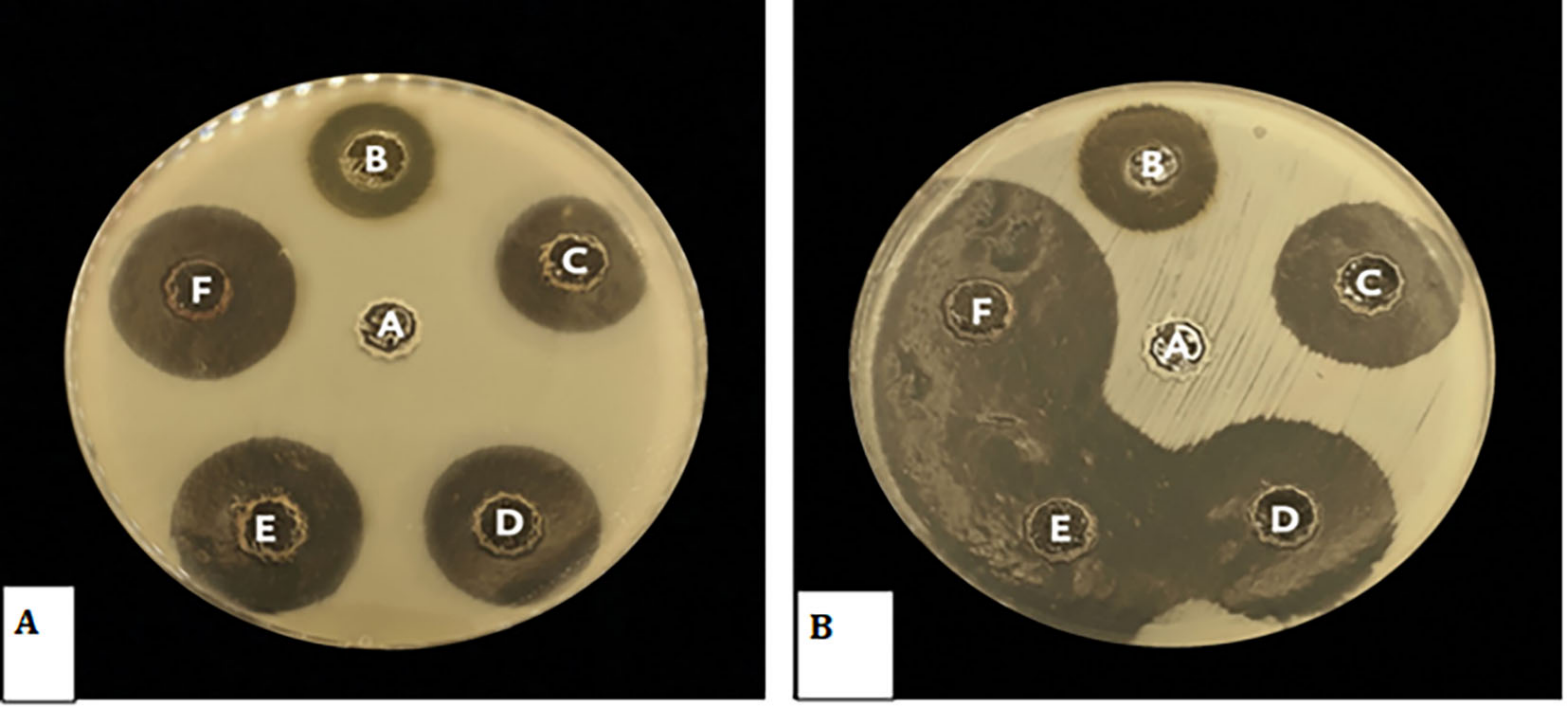
(A) Antibacterial activity of ( *Pelargonium Graveolens* AuNPs) against *Candida Albicans A*, control negative B, Control positive Chlorhexidine 0.12%. C, 0.06 mg/ml. D, 0.12 mg/ml E, 0.25 microgram/ml. F, 0.5 mg/ml (B) Antibacterial activity of ( *Pelargonium Graveolens* AuNPs) against *Streptococcus Mutans,* A, control negative B, Control positive Chlorhexidine 0.12%. C, 0.06 mg/ml. D, 0.12 mg/ml. E, 0.25 microgram/ml. F, 0.5 mg/ml.

**Figure 8.**
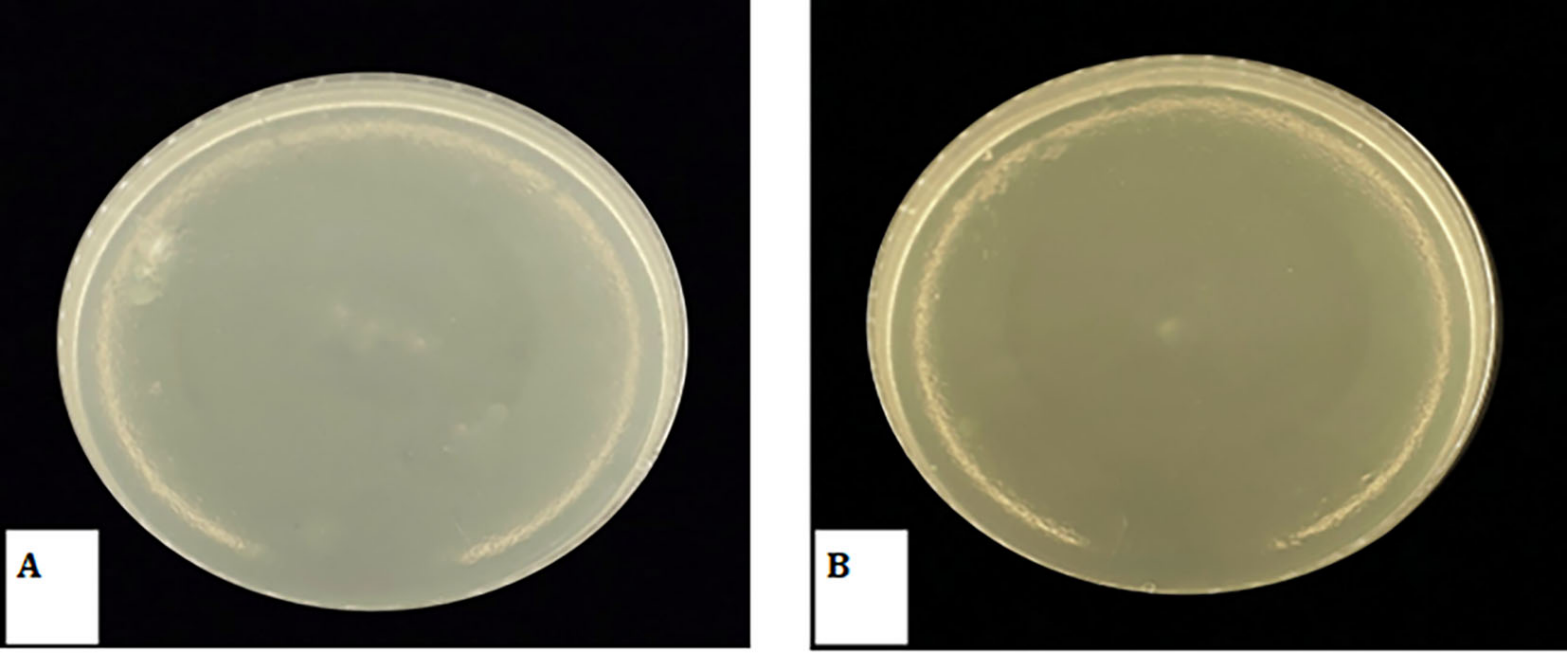
A-MBC against *Streptococcus Mutans, B*- MBC against *Candida albicans*.

The antimicrobial efficacy of *Pelargonium Graveolens* AuNPs was evaluated using *Streptococcus mutans* and *Candida albicans* as test organisms. Significant zones of inhibition were observed subsequent to the exposure of the microorganisms to the *Pelargonium Graveolens* AuNPs that were prepared Figure 7A,B. The findings of the present study demonstrate the effectiveness of the synthesized nanoparticles in inhibiting the growth rate of *Streptococcus mutans* and *Candida albicans* following a 12-hour exposure period as shown in Figure 8.

### Determination of Minimum Bactericidal Concentration (MBC)

The study results indicate that MBC for *Candida albicans* was 0.5 mg/ml, while MBC for *Streptococcus Mutans* was 0.12 mg/ml, as presented in the Tables 3 and 4. Subsequent re-culturing on BHI-agar media revealed no growth at these concentrations as shown in Figure 8 indicating that the 0.5 mg/ml concentration had a fungicidal effect on Candida albicans, while the 0.12 mg/ml concentration had a bactericidal effect on Streptococcus Mutans. Also the results presented in the table indicate that the MIC for Candida albicans was 0.25 mg/ml, while for Streptococcus Mutans it was 0.06 mg/ml. However, onto re-culturing on BHI-agar, it was observed that these concentrations exhibited growth, indicating that they had a bacteriostatic effect.

**Table 3.** Determination of MIC and MBC against *Streptococcus Mutans.*

	Concentration In μg/ml	0.03	0.06	0.12	0.25	0.5	1
*Streptococcus mutans*	Growth	+	-	-	-	-	-

Minimum Inhibitory Concentration (MIC) = 0.06 mg/ml; Minimum Bactericidal Concentration (MBC) = 0.12 mg/ml.

**Table 4.** Determination of MIC and MBC against *Candida albicans.*

	Concentration In μg/ml	0.03	0.06	0.12	0.25	0.5	1
*Candida albicans*	Growth	+	+	+	-	-	-

Minimum Inhibitory Concentration (MIC) = 0.25 mg/ml; Minimum fungicidal Concentration (MBC) = 0.5 mg/ml.

## Discussion

**Figure 9.**
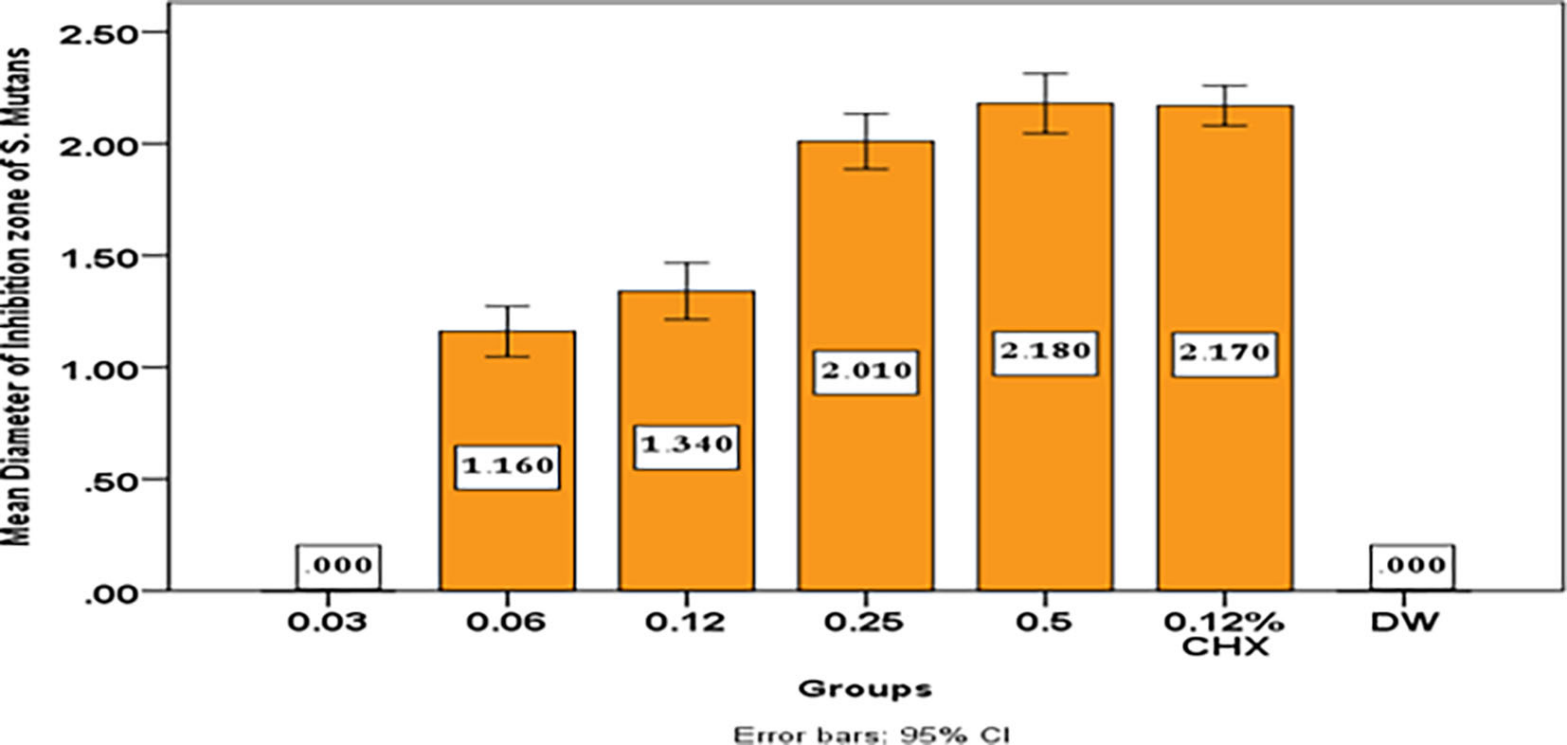
Mean diameter on inhibition zone of *streptococcus mutans.*

The results show that with increase concentration of *Pelargonium Graveolens* AuNPs, the diameter of inhibition zone of streptococcus *mutans* growth increased to reach the maximum diameter as shown in Table 5 and Figure 9.

**Table 5.** Descriptive and statistical test of DIZ of S. Mutans among groups.

Groups	N	Mean	±SD	±SE	Min	Max	F	P value
0.03	10	.0000	.00000	.00000	.00	.00	461.895	0.000 Sig.
0.06	10	1.1600	.15776	.04989	.90	1.50		
0.12	10	1.3400	.17764	.05617	1.00	1.60		
0.25	10	2.0100	.17288	.05467	1.80	2.30		
0.5	10	2.1800	.18738	.05925	1.90	2.40		
0.12% CHX	10	2.1700	.12517	.03958	2.00	2.30		
DW	10	.0000	.00000	.00000	.00	.00		

**Figure 10.**
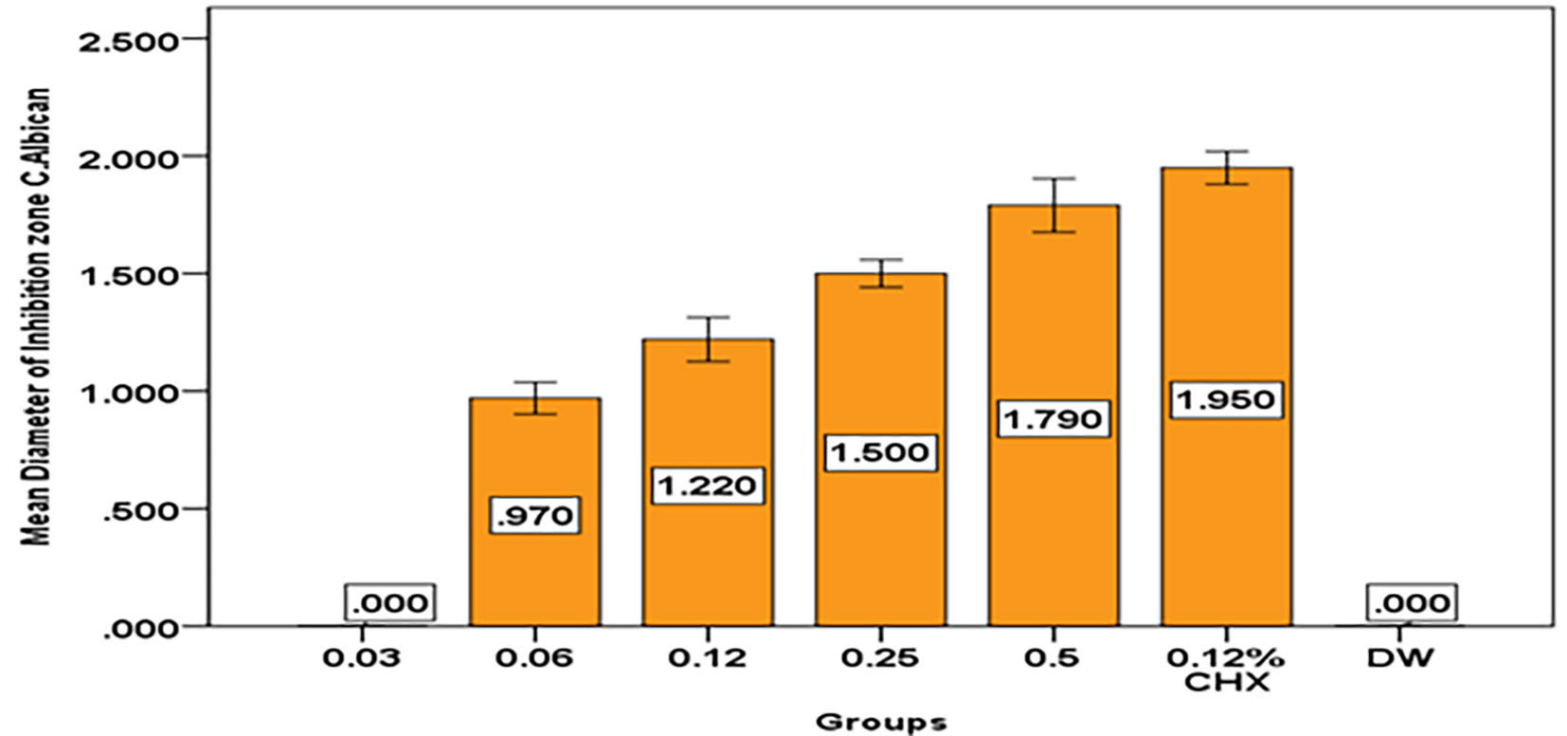
Mean diameter on inhibition zone of *Candida Albican*.

And the results show that with increase concentration of *Pelargonium Graveolens* AuNPs, the diameter of inhibition zone of Candida *Albicans* growth increased to reach the maximum diameter as shown in Table 6 and Figure 10.

**Table 6.** Descriptive and statistical of DIZ of *Candida Albican* among groups.

Groups	N	Mean	±SD	±SE	Min	Max	F	P value
0.03	10	.00000	.000000	.000000	.000	.000	652.939	0.000 Sig.
0.06	10	.97000	.094868	.030000	.800	1.100		
0.12	10	1.22000	.131656	.041633	1.000	1.400		
0.25	10	1.50000	.081650	.025820	1.400	1.600		
0.5	10	1.79000	.159513	.050442	1.500	2.000		
0.12% CHX	10	1.95000	.097183	.030732	1.800	2.100		
DW	10	.00000	.000000	.000000	.000	.000		

In this study although the mean inhibition zone for Streptococcus mutans growth by *Pelargonium Graveolens* AuNPs (1.16 cm) in concentration 0.06 mg/ml is lower than that of Chlorhexidine gluconate 0.12% (2.17 cm), but this mean inhibition zone for Streptococcus mutans growth by *Pelargonium Graveolens* AuNPs significantly increases with increasing concentration till it reaches its highest value 2.18 cm at a 0.5 mg/ml concentration as shown in Table 5 and Figure 9. The study found that *Pelargonium Graveolens* AuNPs at a concentration of 0.5 mg/ml exhibited a mean inhibition zone of 1.79 cm for Candida Albican growth, which was lower than the mean inhibition zone of 1.95 cm, observed for Chlorhexidine gluconate 0.12%. However, it is possible that the mean inhibition zone for Candida Albican growth by *Pelargonium Graveolens* AuNPs may increase with higher concentrations as shown in Table 6 and Figure 10. This finding indicates that the antibacterial effect of *Pelargonium Graveolens* AuNPs is concentration dependent. Due to there is no previous studies on the effect of *Pelargonium Graveolens* AuNPs against *Streptococcus mutans* and *Candida albicans,* so that the study compared with other studies of the effects *Pelargonium Graveolens* AuNPs or effect of the secondary metabolites of *Pelargonium Graveolens* on others microorganisms. This finding may be attributed to the synergistic or additive effect of the secondary metabolites that have antimicrobial activity such as citronellol that have antimicrobial and anti-oxidant activity, ^28^ and the research conducted by Guimares et al., 2019 discovered that citronellol exhibited significant bactericidal properties against S. aureus strains. ^29^ And it agree with Friedman et al., 2004 have classified essential oils and their compounds into two categories, namely slow-acting and fast-acting compounds. The authors have reported that terpineol, eugenol, geranial, carveol, and citronellol are categorized as fast-acting compounds due to their ability to rapidly inactivate microorganisms such as E. coli and S. Typhimurium within a short duration of 2 hours. According to reports, certain antimicrobial agents such as carvacrol, cinnamaldehyde, and geraniol have been identified as fast-acting compounds capable of inactivating microorganisms such as E. coli and S. Typhimurium within a five-minute timeframe. ^30^ Geraniol and citronellol are an active component of *Pelargonium Graveolens.* ^28^ Additionally it may be due to the presence of certain compounds in *Pelargonium Graveolens* AuNPs which might exert their effect either on the cell membrane of streptococci mutans or on the enzymes that are necessary for the growth of this bacteria and this assumption need further studies for its confirmation.

## Conclusion

To our knowledge, the current investigation represents a novel contribution to the scientific literature, as it is the first to demonstrate the antimicrobial efficacy of *Pelargonium Graveolens* synthesized through green synthesis techniques against Streptococcus Mutans and Candida Albicans. There exists a notable degree of interest in the investigation of potential biomedical applications associated with gold nanoparticles (Au NPs). The current investigation employed extracts of *Pelargonium Graveolens* for creating diverse shapes and dimensions of Au NPs. The successful synthesis of stable AuNPs has been supported by a range of characterization methods, including UV-Vis, Zeta potential, FESEM, EDX, XRD, TEM, and FTIR. The composition consists of a combination of hexagonal, spherical, semi-spherical, and triangular like particles nanoparticles with a particle size mean 294nm and low aggregation degree were obtained, exhibiting long-term stability. These nanoparticles can readily associate with diverse bioactive compounds. A positive zeta potential value indicates favorable stability of the particles in the suspension. In addition, it is possible for Au NPs to combine with diverse bioactive compounds that adhere to the *Pelargonium Graveolens* extract, such as protein-based molecules, alkynes and phenolic compounds. This amalgamation could potentially enhance the biological activity of Au NPs. The *Pelargonium Graveolens* AuNPs that were synthesized using green methods have demonstrated effective antibacterial properties against Streptococcus Mutans and Candida Albicans in comparison with Chlorhexidine, while also maintaining a good level of safety. According to these results it can be considered as promising oral health care product in the future and it can be used as mouthwash as alternative of Chlorhexidine mouthwash that still had adverse effects over time.

## Author contributions

conceptualization: Ahmed Yousif Mahdi, Aseel Haidar M.J. Al. Haidar

data curation: Ahmed Yousif Mahdi, Aseel Haidar M.J. Al. Haidar

formal analysis: Ahmed Yousif Mahdi

funding acquisition: Ahmed Yousif Mahdi, Aseel Haidar M.J. Al. Haidar

investigation: Ahmed Yousif Mahdi, Aseel Haidar M.J. Al. Haidar

methodology: Ahmed Yousif Mahdi, Aseel Haidar M.J. Al. Haidar

project administration Ahmed Yousif Mahdi, Aseel Haidar M.J. Al. Haidar

resources: Ahmed Yousif Mahdi, Aseel Haidar M.J. Al. Haidar

software: Ahmed Yousif Mahdi, Aseel Haidar M.J. Al. Haidar

supervision: Aseel Haidar M.J. Al. Haidar

validation: Ahmed Yousif Mahdi, Aseel Haidar M.J. Al. Haidar

visualization: Ahmed Yousif Mahdi, Aseel Haidar M.J. Al. Haidar

Writing – original draft preparation: Ahmed Yousif Mahdi and Aseel Haidar M.J. Al. Haidar

Writing – review and editing: Ahmed Yousif Mahdi, Aseel Haidar M.J. Al. Haidar

## Ethical approval

All of the individuals were given thorough information about the study and the procedures involved, and their informed consent was acquired on a form approved by the ethics committee of the University of Baghdad, College of Dentistry (No. 771233, 26-1-2023, with ref. number 771).

Patients were included in this study after signing an informed consent.

## Data Availability

Raw data for [Green synthesis of gold nanoparticles using Pelargonium Graveolens leaf extract: Characterization and anti-microbial properties (An in-vitro study)], https://doi.org/10.6084/m9.figshare.25674225.v8. ^31^ Data are available under the terms of the Creative Commons Zero “No rights reserved” data waiver (CC0)
