# Supplementary material for: Green synthesis of gold nanoparticles using Pelargonium Graveolens leaf extract: characterization and anti-microbial properties (An in-vitro study)
Source: F1000Res. 2024 Sep 2;13:572. Originally published 2024 Jun 4. [Version 2] doi: 10.12688/f1000research.150769.2 (PMC11350326; doi:10.12688/f1000research.150769.2)
Supplement: Peer review history

# Reviewer report: Reviewer response for version 2

**DOI:** 10.5256/f1000research.171144.r319899

**Published:** 2024-09-03

**Authors:** Saraswathi Jaggali

**Affiliations:** 1 Osmania University, Hyderabad, Telangana, India

The author needs to revise the article thoroughly and make changes where required. For plants, The species and genus should be in Italics, and the genus should be in small letters ( *Pelargonium graveolens or P.graveolens)*. **The same should be true for microbial strains and Figure 7 (B) is not good and  it can be remove other than this article is good.

Is the work clearly and accurately presented and does it cite the current literature?

Yes

If applicable, is the statistical analysis and its interpretation appropriate?

Partly

Are all the source data underlying the results available to ensure full reproducibility?

Yes

Is the study design appropriate and is the work technically sound?

Yes

Are the conclusions drawn adequately supported by the results?

Yes

Are sufficient details of methods and analysis provided to allow replication by others?

Yes

Reviewer Expertise:

Traditional medicines, Pharmacology, Genetics, Molecular Biology, Plant and Animal biotechnology, Bioinformatics drug designing.

I confirm that I have read this submission and believe that I have an appropriate level of expertise to confirm that it is of an acceptable scientific standard.

# Reviewer report: Reviewer response for version 1

**DOI:** 10.5256/f1000research.165366.r315545

**Published:** 2024-08-27

**Authors:** Saraswathi Jaggali

**Affiliations:** 1 Osmania University, Hyderabad, Telangana, India

Research article on Green synthesis gold nanoparticles from *P. graveolens* and their antimicrobial activity is exciting work.

 First, the author must change the microbial strains and plant name in italics form where it is mentioned.

 Second, Previous literature work is given in the form of a reference number but it should be in author-name.

 Statistical analysis P value should be written in the symbol form P>0.05 or P<0.05.

Is the work clearly and accurately presented and does it cite the current literature?

Yes

If applicable, is the statistical analysis and its interpretation appropriate?

Partly

Are all the source data underlying the results available to ensure full reproducibility?

Yes

Is the study design appropriate and is the work technically sound?

Yes

Are the conclusions drawn adequately supported by the results?

Yes

Are sufficient details of methods and analysis provided to allow replication by others?

Yes

Reviewer Expertise:

Traditional medicines, Pharmacology, Genetics, Molecular Biology, Plant and Animal biotechnology, Bioinformatics drug designing.

I confirm that I have read this submission and believe that I have an appropriate level of expertise to confirm that it is of an acceptable scientific standard.

# Response

**Published:** 2024-08-27

**Authors:** Ahmed Asker

**Affiliations:** Pediatric and Preventative Dep, University of Baghdad, college of Dentistry, Baghdad, Baghdad Government, Iraq

Thanks Dr. Saraswathi Jaggali for reviewing my manuscript.

 I am very grateful and I agree with all your comments and revisions.

 Warm regards,

 Ahmed Yousif Mahdi Asker

# Reviewer report: Reviewer response for version 1

**DOI:** 10.5256/f1000research.165366.r288743

**Published:** 2024-06-18

**Authors:** Raya Abid

**Affiliations:** 1 Ibnsina university of medical and pharmaceutical sciences, Baghdad, Iraq

The article is scientific and original. It's necessary to find new approaches in the prevention of diseases and the research was trying to find these approaches.

 Some minor revisions are necessary before the duplication as follow:

-   Abbreviations should  be removed from abstract In abstract.
- Always starting should be done by capital letters. 
- Reference for the isolation and preparation of microorganisms should be added.
- In method,  MIC and other abbreviations  are not clear.
- It's necessary to check the grammar and not use the pronoun 'we'. 
- Its necessary not to start with abbreviations like FTIR when you say previous studies, you have to put them and not just one.
- Finally summarize the conclusion should be more clear and precise.....best regard 

Is the work clearly and accurately presented and does it cite the current literature?

Yes

If applicable, is the statistical analysis and its interpretation appropriate?

Yes

Are all the source data underlying the results available to ensure full reproducibility?

Yes

Is the study design appropriate and is the work technically sound?

Yes

Are the conclusions drawn adequately supported by the results?

Yes

Are sufficient details of methods and analysis provided to allow replication by others?

Yes

Reviewer Expertise:

preventive dentistry

I confirm that I have read this submission and believe that I have an appropriate level of expertise to confirm that it is of an acceptable scientific standard.

# Response

**Published:** 2024-06-23

**Authors:** Ahmed Asker

**Affiliations:** Pediatric and Preventative Dep, University of Baghdad, college of Dentistry, Baghdad, Baghdad Government, Iraq

Thanks Dr. Raya Abid for reviewing my manuscript.

 I am very grateful and I agree with all your comments and revisions.

 Warm regards,

 Ahmed Yousif Mahdi Asker

# Response

**Published:** 2024-06-23

**Authors:** Ahmed Asker

**Affiliations:** Pediatric and Preventative Dep, University of Baghdad, college of Dentistry, Baghdad, Baghdad Government, Iraq

Dear Dr. Raya Abid,

**Below are the corresponding author comments with respect to your review report:**

 1. Ok . I’ll change the abbreviation ( AuNPs ) that appear in the abstract, by (gold nanoparticles). 

 2. Ok. I’ll return to my article and will starting by capital letters. 

 3. The Reference for the isolation and preparation of microorganisms is ( Baron E, Peteson L, Fingold S. Methods for Testing Antimicrobial Effectiveness. In: Bailey and Scotts diagnostic Microbiology. 9th ed. C.V. Mosby Co., St. Louis, USA; 1994.) .

 4. In method the abbreviations are ,  MIC ( minimum inhibitory concentration), MBC (minimum bactericidal concentration) and MFC ( minimum fungicidal concentration), MH-A ( Mueller-Hinton agar ) .

 5. Ok dr. I’ll check all the grammar and not use the pronoun 'we'. 

 6. Ok dr. I’ll  replace the FTIR with Fourier Transform Infrared. 

 7. Thanks Doctor. I’ll summarize the conclusion to be should be more clear and precise as following:- 

 Conclusion 

 (The current investigation employed extracts of Pelargonium Graveolens for creating diverse shapes and dimensions of Au NPs. The successful synthesis of stable AuNPs has been supported by a range of characterization methods, including UV-Vis, Zeta potential, FESEM, EDX, XRD, TEM, and FTIR. The green synthetic gold nanoparticles form Pelargonium Graveolens leaves extract show low aggregation degree were obtained, exhibiting long-term stability. These nanoparticles can readily associate with diverse bioactive compounds. In addition, it is possible for Au NPs to combine with diverse bioactive compounds that adhere to the Pelargonium Graveolens extract, such as protein-based molecules, alkynes and phenolic compounds. This amalgamation could potentially enhance the biological activity of Au NPs that demonstrated effective antibacterial properties against Streptococcus Mutans and Candida Albicans in comparison with Chlorhexidine, while also maintaining a good level of safety. According to these results it can be considered as promising oral health care product in the future and it can be used as mouthwash as alternative of Chlorhexidine mouthwash that still had adverse effects over time.)
