# Supplementary material for: Green synthesis of gold nanoparticles using Pelargonium Graveolens leaf extract: characterization and anti-microbial properties (An in-vitro study)
Source: F1000Res. 2024 Jun 4;13:572. [Version 1] doi: 10.12688/f1000research.150769.1 (PMC11350326; doi:10.12688/f1000research.150769.1)
Supplement: Peer review history

# Response

**Published:** 2024-06-23

**Authors:** Ahmed Asker

**Affiliations:** Department of Pediatric and Pr, University of Baghdad, Baghdad, Baghdad Governorate, Iraq

Thanks Dr. Raya Abid for reviewing my manuscript.

 I am very grateful and I agree with all your comments and revisions.

 Warm regards,

 Ahmed Yousif Mahdi Asker

# Response

**Published:** 2024-06-23

**Authors:** Ahmed Asker

**Affiliations:** Department of Pediatric and Pr, University of Baghdad, Baghdad, Baghdad Governorate, Iraq

Dear Dr. Raya Abid,

**Below are the corresponding author comments with respect to your review report:**

 1. Ok . I’ll change the abbreviation ( AuNPs ) that appear in the abstract, by (gold nanoparticles). 

 2. Ok. I’ll return to my article and will starting by capital letters. 

 3. The Reference for the isolation and preparation of microorganisms is ( Baron E, Peteson L, Fingold S. Methods for Testing Antimicrobial Effectiveness. In: Bailey and Scotts diagnostic Microbiology. 9th ed. C.V. Mosby Co., St. Louis, USA; 1994.) .

 4. In method the abbreviations are ,  MIC ( minimum inhibitory concentration), MBC (minimum bactericidal concentration) and MFC ( minimum fungicidal concentration), MH-A ( Mueller-Hinton agar ) .

 5. Ok dr. I’ll check all the grammar and not use the pronoun 'we'. 

 6. Ok dr. I’ll  replace the FTIR with Fourier Transform Infrared. 

 7. Thanks Doctor. I’ll summarize the conclusion to be should be more clear and precise as following:- 

 Conclusion 

 (The current investigation employed extracts of Pelargonium Graveolens for creating diverse shapes and dimensions of Au NPs. The successful synthesis of stable AuNPs has been supported by a range of characterization methods, including UV-Vis, Zeta potential, FESEM, EDX, XRD, TEM, and FTIR. The green synthetic gold nanoparticles form Pelargonium Graveolens leaves extract show low aggregation degree were obtained, exhibiting long-term stability. These nanoparticles can readily associate with diverse bioactive compounds. In addition, it is possible for Au NPs to combine with diverse bioactive compounds that adhere to the Pelargonium Graveolens extract, such as protein-based molecules, alkynes and phenolic compounds. This amalgamation could potentially enhance the biological activity of Au NPs that demonstrated effective antibacterial properties against Streptococcus Mutans and Candida Albicans in comparison with Chlorhexidine, while also maintaining a good level of safety. According to these results it can be considered as promising oral health care product in the future and it can be used as mouthwash as alternative of Chlorhexidine mouthwash that still had adverse effects over time.)
